# Difference in prostate cancer incidence around sixty years: effects of age and metabolic diseases

**DOI:** 10.1002/cam4.1462

**Published:** 2018-04-25

**Authors:** Jin Bong Choi, Jung Ho Kim, Sung‐Hoo Hong, Kyung‐Do Han, U‐Syn Ha

**Affiliations:** ^1^ Department of Urology Bucheon St. Mary's Hospital College of Medicine The Catholic University of Korea Bucheon Korea; ^2^ Department of Urology Dongnam Institute of Radiological and Medical Sciences Cancer Center Busan Korea; ^3^ Department of Urology Seoul St. Mary's Hospital College of Medicine The Catholic University of Korea Seoul Korea; ^4^ The Cancer Research Institute The Catholic University of Korea Seoul Korea; ^5^ Department of Biostatistics College of Medicine The Catholic University of Korea Seoul Korea

**Keywords:** Age, diabetes mellitus, dyslipidemias, hypertension, metabolic diseases, prostate cancer

## Abstract

We examined the risk of prostate cancer in the Korean population stratified on the basis of age group and risk based on metabolic diseases, using National Health Insurance System (NHIS) data. Of the 51,827,813 people from the NHIS data in 2015, 10,879,591 men without prostate cancer who underwent a health examination were analyzed. The risk of prostate cancer was analyzed with stratification by age. Multivariate‐adjusted Cox regression analysis was conducted to examine the association between prostate cancer and metabolic diseases by age groups. The risk of prostate cancer increased continuously with age and 59 years may be a point of inflection. The hazard ratio (HR) of prostate cancer development rose sharply as that age point passed. The population with metabolic diseases was more likely to develop prostate cancer than the population without any of these components. In addition, the incidence rate ratio (IRR) decreased from the youngest age group to the age group comprising 55–59 year olds. Beyond this age group, there was a plateau. The relative risk for prostate cancer associated with metabolic diseases also showed divergent associations with age. The risk of prostate cancer increased continuously with age and the peak Youden index was at 59 years. The relative risk for prostate cancer according to metabolic diseases also showed divergent associations beyond 59 years of age. Therefore, setting the age threshold at 59 years would improve the present clinical risk stratification for prostate cancer in Korea.

## Introduction

Prostate cancer is one of the most common types of cancer in men worldwide. Although it is less common in the Asia–Pacific region than in the Western Hemisphere, the incidence has increased continuously 1. However, the biological mechanisms linking Westernization and increased prostate cancer are still unclear, age, ethnicity, and family history are demonstrated risk factors 2, 3. Prostate cancer incidence rates also increase with age in the Asia–Pacific region. However, the rate of increase by age groups varies according to the country 1.

In addition to age, multiple epidemiologic studies have suggested that metabolic diseases are associated with cancer including breast, endometrial, ovarian, colorectal, and liver cancer 4, 5. Prostate cancer as well may be linked to metabolic disease. However, the results of individual studies have been inconsistent, and some even reported a negative association of metabolic disease on prostate cancer 6, 7.

We hypothesized that these discrepancies might be due to varying adjustments between studies. Age is an important factor influencing the results, because it is also closely related to metabolic diseases. Therefore, it is important to see how age affects the association between metabolic diseases and the risk of prostate cancer. To our knowledge, there has been no retrospective, large, national cross‐sectional study involving population samples stratified by age groups; therefore, in this study, we examined the risk of prostate cancer in the Korean population stratified by age group and the presence of metabolic diseases using the National Health Insurance System (NHIS) data.

## Materials and Methods

This study used the national health claims database released by the NHIS of Korea. The NHIS offers comprehensive medical care coverage to almost all Koreans, and the remainder of the population is covered by the Medical Aid program 8. In this study, prostate cancer was diagnosed using the International Classification of Diseases, Tenth Revision, Clinical Modification (ICD‐10‐CM) cod C61. Subject name and identification number were anonymized to protect the privacy of patients.

The definition of measurements was also retrieved using ICD‐10‐CM. Hypertension was defined by previous hypertension diagnosis (I10‐13, I15) with a blood pressure ≥140/90 mmHg, or a history of taking antihypertensive drugs. Diabetes was defined by diagnostic codes E10‐14 with a fasting serum glucose level ≥126 mg/dL, or self‐reported use of diabetic drugs. And dyslipidemia was defined by previous diagnostic codes E78 with a total cholesterol level ≥240 mg/dL, or self‐reported use of lipid lowering drugs. Waist circumference (WC) was measured midway between the lower rib margin and the iliac crest in a standing position. The cutoff value of WC for abdominal obesity of men was ≥90 cm 9. Body mass index (BMI) categories, such as underweight (under 18.5), normal weight (18.5–22.9), overweight (23–24.9), and obese (over 25), were from the Korean Society for the Study of Obesity 10. Finally, the definition of patients with metabolic diseases as used in this manuscript refers to individuals with at least one of the common components of metabolic syndrome (diabetes, hypertension, dyslipidemia).

The definition of socioeconomic and lifestyle variables were as follows. Alcohol consumption status was categorized into two groups: non‐to‐moderate drinkers who drank less than two or three times a month and heavy drinkers who drank weekly. Smoking status was also categorized into nonsmokers and ever‐smokers. People were considered to be doing regular exercise if they answered yes to the question, “Did you exercise (running, cycling, hiking, etc.) for over 20 min until you were almost out of breath during the past week?” Income was calculated as the family income that adjusted for the number of family members and divided into low, mid, and high groups.

**Figure 1:**
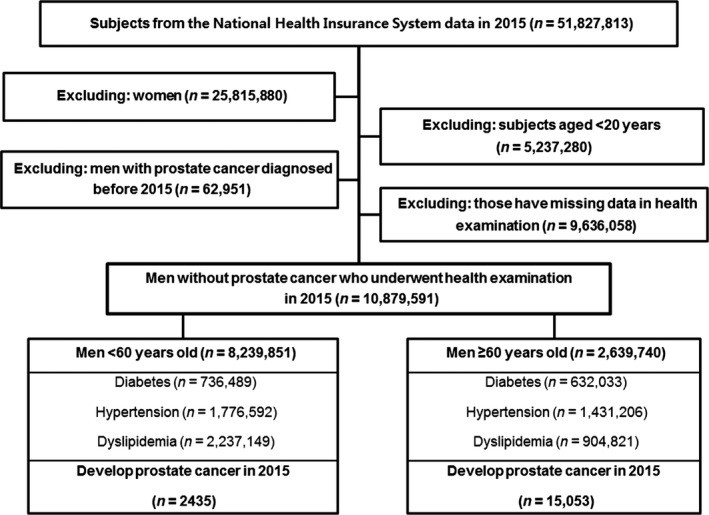
Study design and disposition of subjects.

This study was approved by the Institutional Review Board of the Catholic University of Korea (KC15RISI0633). Of the 51,827,813 people from the NHIS data in 2015, women (*n* = 25,815,880) and those aged <20 years (*n* = 5,237,280) were excluded because prostate cancer is rare in this age group. Information about WC and BMI was reported in health examination databases. After excluding people with prostate cancer diagnosed before January 2015 (*n* = 62,951) and people with data missing on metabolic diseases in health examination databases (*n* = 9,636,058), 10,879,591 men without prostate cancer who underwent health examinations in 2015 were analyzed. A flow of subjects through the study is presented in Figure 1.

For statistical analysis, SAS version 9.4 (SAS Institute, Cary, NC, USA) was used. The data are presented as the mean ± standard error (SE), and as proportions for continuous or categorical variables. The predictive accuracy of age for prostate cancer was assessed by calculating the c‐index on the basis of the receiver operating characteristics (ROC) curve. The cutoff value was defined as the point having the highest Youden index (sensitivity + specificity−1) 11, 12. Incidence rate ratio (IRR) between the prostate cancer patients with metabolic disease and without metabolic disease was calculated per 100,000 person‐years. Multivariate‐adjusted Cox regression analysis was conducted to examine the hazard ratio (HR) and 95% confidence interval (CI) for the association between prostate cancer and metabolic diseases by age groups. A *P*‐value <0.05 was considered statistically significant.

## Results

### General characteristics according to the age group

Table 1 summarizes the general characteristics of the study population in ten‐year brackets. Among a total of 10,879,591 participants, 17,488 (0.16%) were diagnosed with prostate cancer. As expected, the diagnosed age of prostate cancer mainly distributed in older age groups. As the men grow older, the development of prostate cancer increased. The increasing trend of prostate cancer risk was particularly dramatic in populations over the age of 60. An estimated 86% of prostate cancer was diagnosed after 60 years of age. And as the men become older, the prevalence rate of dyslipidemia, hypertension, and diabetes also increased and approached almost 50% in the population over 60 years old.

**Table 1:** Baseline demographic and clinical characteristics according to age groups

Age, years	Age group				
	<40	40–49	50–59	60–69	≥70
No. in population	2,971,221	2,733,742	2,534,888	1,647,426	992,314
No. of diagnosed prostate cancers	43	319	2,073	6,064	8,989
Diabetes					
Yes	74,128 (2.49)	239,789 (8.77)	422,572 (16.67)	382,996 (23.25)	249,037 (25.10)
No	2,897,093 (97.51)	2,493,953 (91.23)	2,112,316 (83.33)	1,264,430 (76.75)	743,277 (74.90)
Hypertension					
Yes	278,135 (9.36)	592,192 (21.66)	906,265 (35.75)	828,523 (50.29)	602,683 (60.74)
No	2,693,086 (90.64)	2,141,550 (78.34)	1,628,623 (64.25)	818,903 (49.71)	389,631 (39.26)
Dyslipidemia					
Yes	307,117 (10.34)	598,595 (21.90)	731,437 (28.85)	568,344 (34.50)	336,477 (33.91)
No	2,664,104 (89.66)	2,135,147 (78.10)	1,803,451 (71.15)	1,079,082 (65.50)	655,837 (66.09)
BMI ≥25 kg/m^2^					
Yes	1,212,651 (40.81)	1,202,974 (44.00)	1,030,226 (40.64)	624,542 (37.91)	298,083 (30.04)
No	1,758,570 (59.19)	1,530,768 (56.00)	1,504,662 (59.36)	1,022,884 (62.09)	694,231 (69.96)
WC ≥90 cm					
Yes	654,484 (22.03)_	662,292 (24.31)	628,762 (24.87)	461,533 (28.06)	284,832 (28.74)
No	2,316,737 (77.97)	2,071,450 (75.77)	1,906,126 (75.20)	1,185,893 (71.98)	707,482 (71.30)
Regular exercise					
Yes	1,939,704 (65.28)	1,731,489 (63.34)	1,557,519 (61.44)	929,931 (56.45)	452,701 (45.62)
No	1,031,517 (34.72)	1,002,253 (36.66)	977,369 (38.56)	717,495 (43.55)	539,613 (54.38)
Smoking status					
Never	1,037,871 (34.93)	268,384 (24.08)	688,342 (27.15)	554,169 (33.64)	456,882 (46.04)
Have ever smoked	1,933,350 (65.07)	2,075,358 (75.92)	1,846,546 (72.85)	1,093,257 (66.36)	535,432 (53.96)
Alcohol consumption status					
≤2~3/month	2,645,865 (89.05)	658,384 (24.08)	688,342 (27.15)	554,169 (33.64)	456,882 (46.04)
≥1/week	325,356 (10.95)	362,997 (13.28)	317,537 (12.53)	146,246 (8.88)	55,578 (5.6)
Household income					
Low	449,695 (15.13)	455,588 (16.67)	554,084 (21.86)	471,160 (28.6)	216,796 (21.85)
Mid	2,163,379 (72.81)	1,405,472 (51.41)	1,145,770 (45.2)	815,306 (49.49)	409,343 (41.25)
High	358,147 (12.06)	872,682 (31.92)	835,034 (32.94)	360,960 (21.91)	366,175 (36.9)

BMI, body mass index; WC, Waist circumference.

Data are presented as the number (%).

### Cutoff value of age for the prediction of prostate cancer

**Figure 2:**
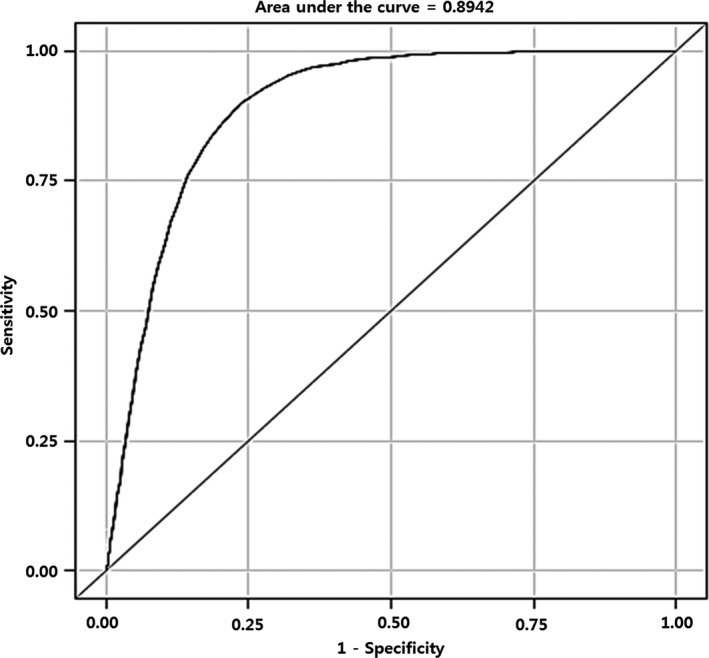
Receiver operating characteristic curve of age in predicting prostate cancer.

The sensitivity, specificity, and Youden index in predicting the development of prostate cancer for different age cutoff values are shown in Table 2. Figure 2 shows the ROC curve on the basis of age for the prediction of prostate cancer. The age at the highest Youden index was 59. The area under the ROC curve for age was 0.8942, and the cutoff value for age was identified on the basis of the highest Youden index.

**Table 2:** Sensitivity and specificity in predicting prostate cancer on the basis of different age cutoffs

Cut off value of age, years	Sensitivity	Specificity	Youden's index^a^
45	0.99097	0.42476	0.41573
46	0.98971	0.44056	0.43027
47	0.98548	0.47578	0.46126
48	0.98376	0.49035	0.47411
49	0.9797	0.52154	0.50124
50	0.9773	0.53728	0.51458
51	0.97204	0.56812	0.54016
52	0.96786	0.58311	0.55097
53	0.95728	0.61478	0.57206
54	0.95128	0.631	0.58228
55	0.93367	0.66563	0.5993
56	0.92458	0.68032	0.6049
57	0.90342	0.71255	0.61597
58	0.89273	0.72529	0.61802
59	0.86328	0.75584	0.61912
60	0.85081	0.76631	0.61712
61	0.8141	0.79548	0.60958
62	0.80369	0.80234	0.60603
63	0.75635	0.82897	0.58532
64	0.74674	0.83428	0.58102
65	0.69379	0.85622	0.55001
66	0.68127	0.86091	0.54218
67	0.60704	0.88514	0.49218
68	0.59664	0.88856	0.4852
69	0.51704	0.90877	0.42581
70	0.50818	0.91114	0.41932

a Youden's index: Sensitivity + Specificity−1.

### Risk of prostate cancer in the population stratified on the basis of age group

Table 3 shows the relative risk of prostate cancer stratified by age group at intervals of 5 years. We set 55–59 years of age as a reference, which is the age range including the highest Youden index. The risk of prostate cancer increased continuously from younger to older age groups during the follow‐up period and in particular the incremental risk increased sharply for populations older than 60 years.

**Table 3:** Crude‐ and multivariable‐adjusted hazard ratios for prostate cancer stratified by age groups

Age, years	No. of population	No. of diagnosed prostate cancers (%)	HR (95% confidence interval)	
			Crude HR	Adjusted HR^a^
≤44	4,613,919	158 (0)	0.026 (0.022, 0.03)	0.027 (0.023, 0.032)
45–49	1,222,497	239 (0.02)	0.148 (0.129, 0.169)	0.151 (0.131, 0.173)
50–54	1,394,904	763 (0.05)	0.413 (0.378, 0.451)	0.419 (0.383, 0.457)
55–59	1,095,002	1449 (0.13)	Ref.	Ref.
60–64	979,335	2746 (0.28)	2.122 (1.991, 2.262)	2.1 (1.97, 2.238)
65–69	599,877	3246 (0.54)	4.106 (3.859, 4.369)	4.021 (3.778, 4.279)
70–74	563,072	4575 (0.81)	6.182 (5.827, 6.559)	6.037 (5.687, 6.409)
75–79	250,407	2635 (1.05)	8.026 (7.527, 8.559)	7.841 (7.346, 8.369)
≥80	160,578	1677 (1.04)	7.965 (7.423, 8.547)	7.791 (7.251, 8.371)

a Adjusted for body mass index, exercise, diabetes, hypertension, dyslipidemia, smoking status, and alcohol consumption status.

### Incidence rates and incidence rate ratio for prostate cancer

**Figure 3:**
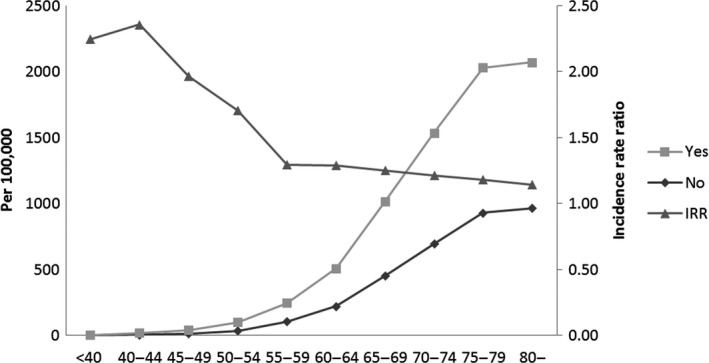
Incidence rate and incidence rate ratio for prostate cancer stratified by age groups between the population with and without metabolic diseases.

Figure 3 shows the incidence rate for prostate cancer and incidence rate ratio (IRR) between populations with and without metabolic diseases. As previously mentioned, the number of new cases of prostate cancer increases with age in the two groups. In addition, it shows differences of incidence rate between the two different populations. Comparing incidence rates, prostate cancer is diagnosed more frequently among populations with metabolic diseases compared with populations without metabolic diseases in all age groups. The IRR was highest in the youngest age group and decreased until about the age with highest Youden index. At older ages IRR exhibited a plateau.

### Risk for prostate cancer according to metabolic diseases across the age groups

Table 4 shows the HR for prostate cancer according to metabolic diseases across the strata of age subgroups. The population with metabolic disease had a higher risk than the population without metabolic disease in all age subgroups. However, divergent associations with regard to age were noted: Those in the youngest cohort of 40–59 years had the highest HR, followed by those aged 60–74 years, and the group who were ≥75 years had the lowest HR. A statistically significant trend of decreasing HR across age subgroups was found in both crude‐, and multivariate‐adjusted models (*P* for interaction <0.001). However, the cumulative incidence markedly increased with age in both populations (Fig. 3).

**Table 4:** Crude‐ and multivariate‐adjusted hazard ratios for prostate cancer according to metabolic disease: stratified by age groups

Age, years	Metabolic disease	No. of population	No. of diagnosed prostate cancers (%)	HR (95% confidence interval)	
				Crude HR	Adjusted HR^a^
All ages	No	7,680,797	6360 (0.08)	Ref.	Ref.
	Yes	3,198,794	11,128 (0.35)	4.212 (4.085, 4.344)	1.368 (1.324, 1.413)
≤59	No	1,360,363	1377 (0.02)	Ref.	Ref.
	Yes	781,921	1232 (0.07)	3.557 (3.294, 3.841)	1.431 (1.321, 1.55)
60–74	No	6,652,105	3668 (0.41)	Ref.	Ref.
	Yes	1,674,217	6899 (0.55)	1.339 (1.286, 1.394)	1.236 (1.187, 1.288)
≥75	No	138,523	1315 (0.95)	Ref.	Ref.
	Yes	272,462	2997 (1.1)	1.16 (1.087, 1.239)	1.141 (1.068, 1.219)
*P* interaction				<0.001	<0.001

a Adjusted for age, body mass index, exercise, smoking status, and alcohol consumption status.

## Discussion

The main findings of this population‐based study are as follows: (1) The risk of prostate cancer increased continuously with age; (2) the age of 59 years (with highest Youden index) may be a point of inflection. The HR of prostate cancer development rose sharply as that point was passed; (3) The population with at least one of the common components of metabolic syndrome (diabetes, hypertension, or dyslipidemia) was more likely to develop prostate cancer than populations without any component. However, there was a definite difference in IRR between less than 55–59 years of age and older. IRR decreased from the youngest group (40–45) to ages 55–59 and thereafter showed a plateau; and (4) The relative risk for prostate cancer coincident with metabolic diseases also showed divergent associations with age. The relative risk for prostate cancer in those with metabolic diseases significantly decreased across the age subgroups, although the cumulative incidence markedly increased with age in either the population with or without metabolic diseases.

Prostate cancer has always been a disease of older men, and age‐specific rates generally rose in a stepwise fashion with peak incidence usually occurring among the oldest age group in a number of populations. However, the trends in age specific incidence rate of prostate cancer had distinct patterns in relation to geographic and ethnic variation 1. In most Western countries including North America and Western Europe, the recent age‐specific incidence rate peaked in a relatively younger age group (65–74) and declined beyond the age of 75 13. In our analysis, the acceleration in rising rates distinctively occurred across the sixties. The HR of prostate cancer development in the late 70s and over 80s increased about eightfold in comparison with HR in the 55–59 age group.

Environmental risk factors can also contribute to prostate cancer. The most important altered environmental risk factor for prostate cancer is that Korea is rapidly becoming an aging society due to increasing life span. The other major factor is the Western diet and life style including high intake fat and calories 14. The Korea National Health and Nutrition Examination Survey (KNHANES) 2015 reported that the prevalence of dyslipidemia sharply increased from 8.0% in 2005 to 17.9% in 2015 15. And consequently the prevalence of obesity increased from 26.2% in 1998 to 39.7% in 2015. These changes have raised concerns about the risk of prostate cancer.

This population‐based cohort study emphasizes two important points. One is that prostate cancer in the younger population (under the age of 60) accounts for a considerable proportion (14.9%) in the Korean population. It has been generally known that Asia has the lowest annual age‐specific incidence rate in comparison with Western nations 13 and old age (over 60s) account for the greater part of newly diagnosed prostate cancer 1. The recent incidence trend which has been extended to younger age groups is worthy of notice. This trend has been influenced by a wide application of prostate‐specific antigen (PSA) testing. Actually, we find geographical incidence differences according to different levels of PSA testing. The mass population‐based prostate cancer screening programs in Austria and Lithuania coincided with the most rapid increases in overall and age‐specific incidence rates 16, 17. In contrast in the United Kingdom 18 and Netherlands 19 where PSA testing rate remains low, prostate cancer incidence rates increased more gradually. In South Korea, PSA screening is common and widespread because the cost of PSA is very low and covered by the national insurance service for all men over 40 years old. Many people receive an annual medical checkup, which includes routine PSA screening.

The other point is that populations with at least one of the common components of metabolic syndrome such as diabetes, hypertension, and dyslipidemia showed clearly higher incidence rates than those without metabolic diseases. It is interesting to find changing effects of metabolic diseases as one gets older. In our report, IRR dropped sharply beyond the age of the highest Youden index and the relative risk also decreased with age in populations with at least one of the common components of metabolic syndrome. These components have recently been considered as possible risk factors for prostate cancer, although the epidemiologic evidence is still conflicted and insufficient to draw any strong conclusion. Moreover, there may be different or even opposing effects of individual components of metabolic syndrome on prostate cancer development. With respect to diabetes, some recent studies provided reports favoring a protective effect in Caucasians 20, 21, Iranians 22, Israelis 23, African Americans, Native Hawaiians, and Japanese Americans 20. In contrast, studies conducted on Taiwanese 24 and Koreans 25 provided positive links between diabetes and prostate cancer. In particular, it was noteworthy that these proposed potential risk factors may have different effects on the development of prostate cancer by age group. It is partly due to that the elderly are more likely to have been exposed to risk factors for development of cancer such as chronic inflammation, hormone disturbance, immunosuppression, poor nutrition, and other health‐related behaviors than young people 26. These factors could not all be included in this study as adjustment factors. Therefore, the influence of metabolic disease on development of cancer might be relatively small in young people than in older people.

One limitation of this study is that selection bias might have occurred. The population with underlying metabolic diseases is usually more likely to have opportunity to use medical services. Consequently, they have more chance to seek medical advice and be screened for prostate cancer. Likewise, there was a higher opportunity for PSA screening due to evaluation of voiding dysfunction at older ages. However, it may be enough to offset this potential selection bias and increase the reliability of the result that our target population lives where low‐cost PSA testing due to the national health insurance and the annual health examination has been spreading. In addition, the current study is population based with a large nationally representative sample based on the NHIS database, which has considerable strength and power.

## Conclusions

The risk of prostate cancer continuously increased with age, and the age with highest Youden index was 59 years. The relative risk for prostate cancer according to metabolic diseases also showed divergent associations beyond 59 years of age. In other words, the influence of metabolic diseases on the development of prostate cancer was high before 59 years, although the rate of increase with age was low. Therefore, setting the age threshold at 59 years for prostate cancer development would improve the present clinical risk stratification for prostate cancer in Korea.

## Conflict of Interest

The authors declare no competing financial interests.
